# Improving laying hen performance and environmental sustainability: effects of combined bacillus strains on egg quality, gut health, and harmful gas emissions

**DOI:** 10.1016/j.psj.2025.105778

**Published:** 2025-09-03

**Authors:** Wei Han Zhao, Madesh Muniyappan, Shan Chuan Cao, In Ho Kim

**Affiliations:** a Department of Animal Biotechnology, Dankook University, Cheonan, South Korea; b Smart Animal Bio Institute Dankook University, Cheonan, Korea; c Jiangsu Key Laboratory of Animal Genetic Breeding and Molecular Design, College of Animal Science and Technology, Yangzhou University, Yangzhou, China

**Keywords:** Bacillus, Egg production performance, Egg quality, Intestinal morphology, Laying hens

## Abstract

This study evaluated the effects of a compound probiotic preparation consisting of *Bacillus subtilis (B. subtilis)* and *Bacillus licheniformis (B. licheniformis)* (collectively referred to as CBS) on laying hen performance, egg quality, nutrient digestibility, intestinal morphology, antioxidant capacity, cecal microbiota, and excreta gas emissions. A total of 468 Hy-Line Brown laying hens, 24 weeks of age, were randomly allocated to three treatment groups: a basal diet group (CON), a group receiving 0.01 % (4.1 × 10^7^ CFU/g of *B. licheniformis* and 7.0 × 10^7^ CFU/g of *B. subtilis*) CBS (TRT1), and a group receiving 0.02 % CBS (TRT2). Each group included 13 replicates with 12 hens per replicate. The experimental period lasted for 10 weeks. The results showed that dietary CBS supplementation significantly increased egg production during weeks 6 and 7 (*p* < 0.05) and significantly reduced the feed conversion ratio (FCR) during weeks 1, 3, 4, 6, 7, and 8 (*p* < 0.05). Additionally, CBS significantly improved eggshell thickness at week 4, Haugh unit and albumen height at weeks 6 and 8, and eggshell strength at weeks 6 and 10 (*p* < 0.05).

Morphological analysis revealed that CBS increased villus height (VH) in the jejunum and duodenum, elevated the villus height-to-crypt depth (VH/CD) ratio, and reduced crypt depth (CD) in the duodenum, jejunum, and ileum. In terms of serum biochemistry, CBS significantly decreased levels of triglycerides, low-density lipoprotein cholesterol (LDL-C), and malondialdehyde (MDA), while significantly increasing levels of high-density lipoprotein cholesterol (HDL-C), immunoglobulin A (IgA), immunoglobulin G (IgG), superoxide dismutase (SOD), total antioxidant capacity (T-AOC), and glutathione peroxidase (GSH-Px). In liver tissue, CBS supplementation significantly enhanced the activities of SOD, GSH-Px, and catalase (CAT), and significantly lowered hepatic MDA concentrations (*p* < 0.05).

Furthermore, CBS significantly improved the digestibility of dry matter and nitrogen, increased cecal Lactobacillus counts, and decreased the abundance of Escherichia coli. CBS supplementation also significantly reduced excreta emissions of ammonia (NH₃), hydrogen sulfide (H₂S), and acetic acid (*p* < 0.05).

In conclusion, dietary supplementation with 0.02 % CBS effectively improved egg production and egg quality in laying hens, likely due to enhanced antioxidant activity and favorable effects on nutrient digestibility, intestinal morphology, cecal microbiota, and reduction in harmful excreta gas emissions.

## Introduction

In the poultry industry, laying hens often experience suboptimal performance, reduced egg quality, and, in some cases, higher mortality rates. Key indicators of decreased laying performance include a lower egg production rate, poorer egg quality such as a reduced Haugh unit, and an increased feed (Yao et al., 2023). As laying hens age and become increasingly stressed, they often experience declining health. Contributing factors include elevated levels of reactive oxygen species (ROS), disruptions in metabolic homeostasis, and a reduced capacity to combat oxidative stress. In addition, immune homeostasis is compromised, immune activity weakens, and damage to immunocompetent cells becomes more likely (Adam et al., 2024; Arulnathan et al., 2024). Various microbial species reside in the intestinal tract of laying hens and play essential roles in host physiological processes, including respiration, reproduction, and gastrointestinal function (Upadhaya et al., 2019). Several factors associated with poultry management during the laying period, such as damage to the mucosal lining of the gut and reproductive tract and stress-induced immunosuppression, can lead to gastrointestinal and reproductive tract infections. These infections disrupt the composition of the gut microbiota and increase the susceptibility of hens to disease (Burkholder et al., 2008; Oluwagbenga et al., 2023). Age and the laying period significantly affect the microbial composition of laying hens’ intestines. The balance of intestinal flora can be easily disrupted, weakening the digestive system of laying hens and promoting inflammatory responses, both of which reduce production performance (Khan et al., 2020; Dai et al., 2022). To maintain bird health and ensure the safe production of eggs, the ovary, which is the site of egg formation, must be protected against pathogens. Research has shown that higher egg production performance during the mid-laying phase can be linked to immune imbalances, impaired intestinal function, and disruption of intestinal flora caused by intensive production practices (Reis et al., 2023). Due to their unique ability to inhibit pathogens, compete with harmful microbes for adhesion sites, and stimulate, modulate, and regulate the host’s immune system by activating specific genes both inside and outside the intestinal tract, probiotic dietary supplements have demonstrated positive effects against a variety of enteric pathogens (Prajapati et al., 2024).

Numerous studies have shown that 1 × 10^5^ CFU/g probiotics enhance the productivity and health of laying hens, contributing to their widespread use and promotion in the laying hen industry. (Oladokun et al., 2023; Qin et al., 2024). *Bacillus species*, beneficial organisms found in the intestines of animals, are commonly used to improve growth and intestinal health in both healthy and pathogen-challenged environments (Xu et al., 2021; Zhang et al., 2021; Liu et al., 2023). Additionally, the primary Bacillus species in laying hens have been enhanced over the past several decades (Guo et al., 2018; Chen et al., 2020). *B. licheniformis* and *B. subtilis*, both spore-forming bacteria, remain metabolically inactive to withstand harsh environments (Earl et al., 2008). Among the most widely used probiotics in the poultry industry are *B. licheniformis* and *B. subtilis*, known for their resistance to high temperatures and stress. According to reports, adding Bacillus subtilis at a concentration of 1.0 × 10⁸ CFU/g can improve gut health and enhance overall host health by inhibiting pathogen growth, lowering intestinal pH through acid fermentation, and strengthening the gut-associated immune system. (Guo et al., 2018; Whelan et al., 2019). Previous studies have reported that 1.0 × 10⁸ CFU/g *B. subtilis* can improve gut morphology, increase microbial diversity, and help regulate the balance of gut microbiota in laying hens (Guo et al., 2017; Oh et al., 2017). Furthermore, *B. subtilis*, fermented *B. subtilis* products, or probiotic powder supplements have been shown to enhance feed efficiency and body weight gain (Xu et al., 2006). Previous studies demonstrated that adding 0.05 % *B. subtilis* to laying hens’ diets can effectively reduce the negative effects of heat stress on their performance (Forte et al., 2016b; Fathi et al., 2018). Dietary inclusion of 1 × 10^8^ CFU/kg *B. subtilis* can increase the activity of CAT, GSH-Px, and SOD, while MDA levels in chickens (Oladokun et al., 2023).Research has shown that 5 × 10^9^ CFU/gm *B. licheniformis* produces various beneficial compounds that support animal performance and promote immune system development, including bacteriocins, digestive enzymes, and antibacterial peptides (Devyatkin et al., 2021). Adding 1.0 × 10^10^ CFU/g *B. licheniformis* to the diet can improve growth performance and nutrient digestibility (Deng et al., 2018). In fattening lambs, dietary supplementation with 6 × 10^9^ CFU/kg *B. licheniformis* increased GSH-Px and SOD activities, decreased ammonia nitrogen (NH_3__—_N) levels, and elevated microbial crude protein (Jia et al., 2018). (Xu et al., 2021) reported that dietary inclusion of both 1.5 × 10^9^ CFU/kg *B. subtilis* and *B. licheniformis* increased CAT, GSH-Px, and SOD levels while decreasing MDA in chickens. Moreover, 1 × 1^7^ CFU/g *B. licheniformis* improved laying performance and egg quality in a dose-dependent manner (Deng et al., 2012).

We hypothesized that including CBS in the diet could improve egg quality, nutrient digestibility, antioxidant capacity, intestinal morphology, and reduce ammonia emissions in laying hens. To our knowledge, no data currently exist on the effects of CBS supplementation in laying hen diets. Therefore, this study aimed to evaluate the impact of CBS inclusion on laying performance, egg quality, nutrient utilization, intestinal morphology, antioxidant capacity, and excreta noxious gas emissions in laying hens.

## MATERIALS AND METHODS

The protocols for the experimental management and care of animals underwent review and were approved (DK-1-2109) by the Animal Care and Use Committee of Dankook University in South Korea.

### Product source

The probiotic mixture used in this study was a commercial product manufactured by GAIA BIO Co., Ltd. (Hwaseong-si, Gyeonggi-do, Republic of Korea). At a supplementation level of 0.01 % in the feed, it was guaranteed to provide Bacillus licheniformis at 4.1 × 10⁷ CFU/g and Bacillus subtilis at 7.0 × 10⁷ CFU/g. According to the manufacturer, after administration, Bacillus licheniformis maintains an activity of 2.2 × 10⁶ CFU/g, and Bacillus subtilis maintains 3.7 × 10⁶ CFU/g.

### Animals, diets, and experimental design

A total of 468 Hy-Line Brown laying hens, 24 weeks of age, were used in this study. The hens were randomly assigned to one of three dietary treatment groups, with 13 replicates per group and 12 hens per replicate. The study included a 7-day adaptation period followed by a 10-week experimental phase. The three dietary treatments were as follows: (1) CON group: basal diet; (2) TRT1 group: basal diet supplemented with 0.01 % CBS; and (3) TRT2 group: basal diet supplemented with 0.02 % CBS. The CBS product consisted of the dry blend *B. licheniformis* and *B. subtilis* spores. The diets were formulated based on the nutritional standards outlined in the Hy-Line Brown Management Guide (2023), with corn and soybean meal as the primary ingredients. Detailed formulations are shown in Table 1. The hens were housed in stainless-steel cages measuring 50 cm x 40 cm x 38 cm. Throughout the 10-week trial, environmental conditions were carefully controlled to minimize external influencing factors. Relative humidity was maintained between 60 % and 70 %. A daily photoperiod of 16 hours light and 8 hours dark was implemented, with lights turned on at 6:00 a.m. and off at 10:00 p.m. Ventilation was regulated by an automatic airflow control system to ensure adequate air exchange and to reduce the accumulation of harmful gases. Room temperature was maintained at 24 ± 1°C and kept consistent until the end of the study. All hens had ad libitum access to feed and water. House conditions were monitored five times daily (06:00, 10:00, 14:00, 18:00, and 22:00) to check feed and water availability as well as bird health.

**Table 1:** Composition of laying hen diets (as fed-basis).

Item	CON
Ingredients (%)	
Corn	54.72
Distillers Dried Grains with Solubles	5.00
Palm kernel meal	8.85
Soybean meal	17.29
Sesame meal	2.00
Tallow	1.22
Mono-di-calcium Phosphate	0.60
Limestone	9.80
Salt	0.05
Methionine (99 %)	0.07
Lysine (50 %)	0.05
Vitamin mix^1^	0.10
Mineral mix^2^	0.10
Choline (50 %)	0.10
Phytase (500unit)	0.05
Total	100.00
Determined value (%)	
Dry Matter	90.39
Moisture	9.61
Crude Protein	16.02
Crude Fat	4.27
Crude Fiber	4.66
Crude Ash	4.57
Calculated value (%)	
Calcium	4.18
Phosphorus	0.94
Available Phosphorus	0.36
Lysine	0.75
Methionine + Cystine	0.91
MEn (kcal/kg)	2560
Linoleic Acid	1.94
Vit D (IU)	1500

1 Provided per kilograms of diet: vitamin A, 10,800 IU; vitamin D3, 4,000 IU; vitamin E, 40 IU; vitamin K3, 4 mg; vitamin B1, 6 mg; vitamin B2, 12 mg; vitamin B6, 6 mg; vitamin B12, 0.05 mg; biotin, 0.2 mg; folic acid, 2 mg; niacin, 50 mg; d-calcium pantothenate, 25 mg.

2 Provided per kg diet: Fe, 100 mg as ferrous sulfate; Cu, 17 mg as copper sulfate; Mn, 17 mg as manganese oxide; Zn, 100 mg as zinc oxide; I, 0.5 mg as potassium iodide; and Se, 0.3 mg as sodium selenite.

### Laying performance and egg quality evaluation

Egg production was recorded daily and expressed as the hen-day egg production rate. Eggs were weighed twice per week on a replicate basis. Egg production rate, average egg weight, average daily feed intake (ADFI), and FCR were calculated weekly. At weeks 2, 4, 6, 8, and 10 of the experiment, 30 eggs were randomly collected from each group for egg quality evaluation at the same time of day (in the morning). Eggshell color was assessed using an eggshell color fan (Samyang Co., Ltd., Seoul, South Korea). Eggshell thickness was measured using a caliper gauge (Ozaki MFG. Co., Ltd., Tokyo, Japan) at the blunt end, pointed end, and middle of the shell, and the average was calculated after removing the shell membrane. Egg weight, Haugh unit, and yolk color were determined using a multifunctional egg quality analyzer (Touhoku Rhythm Co., Tokyo, Japan).

### Serum parameters and antioxidant indices

At the conclusion of the trial, broilers in each treatment group underwent an 8-hour fasting period before blood collection. Thirteen hens were randomly selected from each treatment group. Blood samples (10 mL) were collected from the wing vein of each hen and centrifuged at 3,000 × *g* for 10 minutes at 4°C. The separated serum was stored at –80°C for subsequent analysis. Additionally, 6 g of liver tissue was collected and immediately frozen in liquid nitrogen for the evaluation of antioxidant activity. Levels of SOD, GSH-Px, CAT, T-AOC, and MDA in the serum and liver samples were measured using commercial kits provided by Nanjing Jiancheng Bioengineering Institute (Nanjing, Jiangsu, China). IgA, IgG, and Immunoglobulin M (IgM) levels were also determined using ELISA kits from the same supplier. Serum levels of total cholesterol, triglycerides, HDL cholesterol, and LDL cholesterol were analyzed using a fully automated blood analyzer (Hitachi 7020, Hitachi High-Technologies Corporation, Tokyo, Japan).

### Nutrient digestibility

To evaluate nutrient digestibility, 0.20 % chromium oxide (Cr₂O₃) was added to the diets during the final 7 days of the trial. Excreta were collected daily for 7 consecutive days using trays placed under the cages and stored at –20°C for later analysis. Feed and freeze-dried excreta samples were dried in a forced-air oven at 60°C for 72 hours, then ground using a Wiley mill equipped with a 1 mm screen. Dry matter, nitrogen, and energy contents were determined according to (AOAC, 2010) methods. Chromium concentration was measured using a UV-visible spectrophotometer (Shimadzu, UV-1201, Kyoto, Japan), following the method described by (Williams et al., 1962). The following formula was used to calculate the apparent total tract digestibility (ATTD).

$$\mathrm{ATTD}\;\left(\%\right)\;=\;[1\;-\;\left\{\left(N_{f}\times \;C_{d}\right)\;/\;\left(N_{d}\;\times \;C_{f}\right\}\right]\;\times \;100$$

where: N_f_ indicated concentration in excreta (% DM), N_d_ indicated nutrient concentration in diets (% DM), C_f_ indicated chromium concentration in excreta (% DM), and C_d_ indicated chromium concentration in diets (% DM).

### Intestinal morphology

Thirteen hens per treatment group were randomly selected and euthanized by cervical dislocation and exsanguination. Tissue samples from the jejunum, ileum, and duodenum were immediately collected, rinsed with cold saline (9 g/L NaCl, 4°C), and fixed in paraformaldehyde for further analysis. Tissues were sectioned, dried, dehydrated, cleared, and embedded in paraffin. After embedding, sections were cut, stained with hematoxylin and eosin, evaluated for quality, and mounted using neutral gum. Each tissue section was examined under a light microscope and photographed. VH and CD were measured using Image-Pro Plus 6.0 software, and the average of five measurements per sample was used for data analysis.

### Cecal microbial counts

In addition, the left cecum of each of the 13 slaughtered hens per group was collected, sealed in plastic bags, and stored at –18°C. Cecal contents were later thawed and transferred into sterile tubes. Serial dilutions were prepared using 0.85 % sterile saline. A 0.1 mL aliquot of each dilution was plated on MacConkey agar, and de Man, Rogosa, and Sharpe (MRS) agar (Difco Laboratories, Detroit, MI, USA), and incubated at 37°C for 24 hours. After incubation, target colonies were counted and results were expressed as log₁₀ CFU/g.

### Noxious gas emissions from excreta

At the end of the experiment, fresh excreta samples were collected from each pen to evaluate harmful gas emissions. Mixed samples (300 g) were placed in 2.6-L sealed plastic containers and fermented at room temperature (25°C) for 7 days. Following the method of Hossain et al. (2024), a small hole was drilled in the resin-sealed lid of each container. A 100 mL headspace gas sample was collected from a height of 5 cm above the excreta surface using a gas sampling pump and gas detector tubes (GV-100; Gastec Co., Kanagawa, Japan; detector tube numbers: 3 L, 4LT, and 70 L). The concentrations of H₂S, NH₃, total mercaptans, acetic acid, and carbon dioxide (CO₂) were then measured.

### Statistical analysis

All data were analyzed using SAS 9.4 (SAS Institute Inc., Cary, NC, USA). A one-way analysis of variance (ANOVA) was used to identify significant differences among treatment groups. Relationships among blood parameters, egg quality, and cecal microbiota were assessed using Spearman’s correlation test. Cecal microbial counts, intestinal morphology, and serum parameters and antioxidant indices are usually measured at the individual bird level, whereas laying performance, egg quality, noxious gas emissions from excreta, and nutrient digestibility are typically measured at the pen level. For all statistical analyses, a P-value < 0.05 was considered significant.

## Results

### Production performance and egg quality

As shown in Table 2, the inclusion of CBS had no significant effect on ADFI or downgraded egg quality (*p* > 0.05). However, dietary supplementation with CBS significantly reduced FCR (*p* < 0.05) during weeks 1, 3, 4, 6, 7, and 8, and significantly improved egg production (*p* < 0.05) during weeks 6 and 7 compared to the CON group. Additionally, CBS supplementation significantly increased (*p* < 0.05) eggshell thickness at week 4, albumen height and Haugh unit at weeks 6 and 8, and eggshell strength at weeks 6 and 10 (Table 3).

**Table 2:** The effects of dietary CBS supplementation on egg production performance in laying hens^1^.

Items	CON	TRT1	TRT2	SEM	*P*-value
Body weight (g)					
Initial	2076	2076	2077	23.54	0.999
Week 10	2101	2112	2115	25.15	0.918
Week 1					
Downgraded egg (%)	1.077	0.947	0.746	0.399	0.842
Egg production (%)	96.13	96.77	97.42	0.542	0.255
ADFI (g/d/hen)	95.61	95.58	95.23	0.61	0.964
FCR	1.86^a^	1.84^ab^	1.81^b^	0.011	0.005
Week 2					
Downgraded egg (%)	1.12	1.01	0.92	0.337	0.916
Egg production (%)	97.97	98.63	99.54	0.917	0.486
ADFI (g/d/hen)	97.96	96.99	96.74	0.34	0.339
FCR	1.81	1.81	1.77	0.017	0.155
Week 3					
Downgraded egg (%)	1.24	1.05	1.11	0.304	0.907
Egg production (%)	98.43	98.06	98.62	0.672	0.836
ADFI (g/d/hen)	98.37	98.01	97.88	0.24	0.714
FCR	1.83^a^	1.82^ab^	1.79^b^	0.012	0.04
Week 4					
Downgraded egg (%)	1.25	0.93	0.91	0.395	0.788
Egg production (%)	97.79	98.34	98.25	0.810	0.878
ADFI (g/d/hen)	98.86	98.72	98.08	0.36	0.676
FCR	1.83^a^	1.81^ab^	1.78^b^	0.015	0.061
Week 5					
Downgraded egg (%)	1.04	0.95	1.24	0.318	0.803
Egg production (%)	98.08	96.97	98.07	0.986	0.660
ADFI (g/d/hen)	98.46	98.40	96.73	0.36	0.137
FCR	1.82	1.82	1.78	0.019	0.339
Week 6					
Downgraded egg (%)	1.14	0.93	1.12	0.358	0.904
Egg production (%)	96.42	96.42	97.88	0.843	0.037
ADFI (g/d/hen)	97.92	97.80	97.65	0.28	0.931
FCR	1.84^a^	1.82^ab^	1.77^b^	0.017	0.013
Week 7					
Downgraded egg (%)	1.34	1.19	1.08	0.384	0.896
Egg production (%)	96.05	96.14	97.88	0.798	0.020
ADFI (g/d/hen)	97.16	96.98	96.60	0.55	0.934
FCR	1.83^a^	1.81^a^	1.76^b^	0.015	0.005
Week 8					
Downgraded egg (%)	1.33	1.25	1.11	0.473	0.945
Egg production (%)	95.39	96.68	96.58	0.474	0.117
ADFI (g/d/hen)	98.27	97.81	96.89	0.43	0.445
FCR	1.85^a^	1.80^b^	1.78^b^	0.010	<0.001
Week 9					
Downgraded egg (%)	1.30	1.17	1.15	0.346	0.944
Egg production (%)	95.32	96.32	96.59	0.672	0.377
ADFI (g/d/hen)	97.91	97.17	96.89	0.48	0.707
FCR	1.84	1.80	1.80	0.013	0.062
Week 10					
Downgraded egg (%)	1.47	1.10	1.15	0.385	0.766
Egg production (%)	94.75	95.50	95.13	0.649	0.721
ADFI (g/d/hen)	97.02	97.33	97.86	0.41	0.730
FCR	1.85	1.82	1.81	0.012	0.080

Abbreviation: CON, control group; CBS, combination of *B. subtilis* and *B. licheniformis*; FCR, feed conversion ratio; ADFI, average daily feed intake; SEM, standard error of means.

1 Data are means and SEM (*n* = 13). Means followed by the different letters in the same row show a significantly difference (*p* < 0.05).

**Table 3:** The effects of dietary CBS supplementation on egg quality in laying hens^1^.

Items	CON	TRT1	TRT2	SEM	P-value
Week 2					
Egg shell color	10.47	10.80	11.23	0.283	0.165
Haugh unit	97.76	97.94	98.18	1.660	0.812
Egg weight (g)	61.61	61.12	61.94	0.878	0.804
Yolk color	8.77	8.20	8.70	0.257	0.240
Albumen height (mm)	10.63	10.71	11.03	0.564	0.869
Strength (kg/cm^2^)	3.80	3.98	3.84	0.197	0.780
Eggshell thickness (mm^−2^)	36.7	37.4	36.6	0.514	0.490
Week 4					
Egg shell color	12.0	12.36	12.30	0.191	0.356
Haugh unit	93.74	95.31	96.57	2.293	0.684
Egg weight (g)	61.82	63.54	61.90	0.780	0.773
Yolk color	8.67	8.47	8.87	0.228	0.466
Albumen height (mm)	7.67	8.19	8.90	0.422	0.111
Strength (kg/cm^2^)	3.97	4.02	4.46	0.799	0.887
Eggshell thickness (mm^−2^)	37.9^b^	39.4^ab^	39.9^a^	0.542	0.029
Week 6					
Egg shell color	12.17	12.70	12.50	0.196	0.156
Haugh unit	91.43	94.56	95.78	1.720	0.018
Egg weight (g)	61.49	61.55	63.50	1.013	0.287
Yolk color	8.67	8.70	8.60	0.152	0.894
Albumen height (mm)	8.15	9.50	10.44	1.685	0.043
Strength (kg/cm^2^)	3.45^b^	3.90^ab^	4.28^a^	0.168	0.003
Eggshell thickness (mm^−2^)	38.53	40.07	40.0	0.626	0.153
Week 8					
Egg shell color	10.80	11.06	11.03	0.262	0.737
Haugh unit	92.86	95.82	95.43	2.593	0.022
Egg weight (g)	62.68	61.55	62.97	0.772	0.394
Yolk color	8.73	8.50	8.33	0.211	0.407
Albumen height (mm)	7.38^b^	8.21^ab^	9.41^a^	0.539	0.032
Strength (kg/cm^2^)	3.92	4.15	4.28	0.125	0.125
Eggshell thickness (mm^−2^)	39.20	39.57	39.13	0.368	0.670
Week 10					
Egg shell color	11.0	10.97	11.10	0.257	0.930
Haugh unit	95.08	94.88	92.64	2.657	0.772
Egg weight (g)	63.08	63.42	62.00	0.838	0.462
Yolk color	8.33	8.83	8.57	0.192	0.189
Albumen height (mm)	8.06	8.55	8.83	0.557	0.615
Strength (kg/cm^2^)	4.14	4.65	4.65	0.164	0.045
Eggshell thickness (mm^−2^)	39.83	39.00	39.30	0.362	0.263

Abbreviation: CON, control group; CBS, combination of *B. subtilis* and *B. licheniformis*; SEM, standard error of means.

1 Data are means and SEM (*n* = 30). Means followed by the different letters in the same row show a significantly difference (*p* < 0.05).

### Nutrient digestibility

**Fig. 1:**
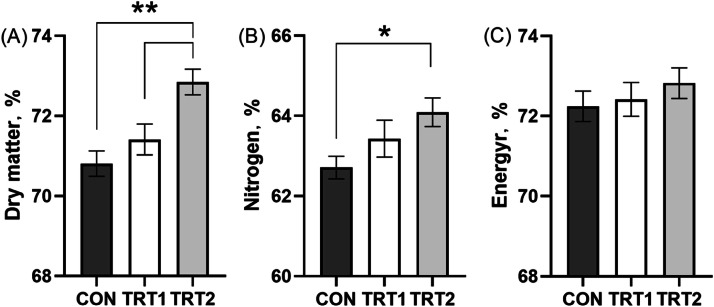
Nutrient digestibility on laying hens. (A) Dry matter. (B) Nitrogen. (C) Energy. CON group, basal diet; TRT 1 group, basal diet+0.01 % combination of *B. subtilis* and *B. licheniformis*; TRT 2 group, basal diet+0.02 % combination of *B. subtilis* and *B. licheniformis*. Values are represented as mean ± SEM, *N* = 8. * denotes *p* < 0.05.

Dietary inclusion of CBS significantly improved dry matter (*p* < 0.01) and nutrient digestibility (*p* < 0.05) compared to the control group, while no significant effect was observed on energy digestibility (Figure 1).

### Immune indices

**Fig. 2:**
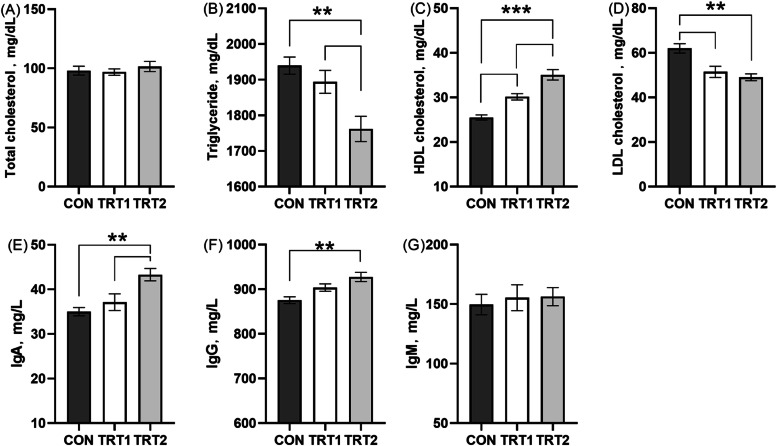
Serum indices of laying hens. (A) Total cholesterol. (B) Triglyceride. (C) High-density lipoprotein cholesterol. (D) Low-density lipoprotein cholesterol. (E) Immunoglobulin A. (F) Immunoglobulin G. (G) Immunoglobulin M. CON group, basal diet; TRT 1 group, basal diet+0.01 % combination of *B. subtilis* and *B. licheniformis*; TRT 2 group, basal diet+0.02 % combination of *B. subtilis* and *B. licheniformis*. Values are represented as mean ± SEM, *N* = 6. * denotes *p* < 0.05, ^⁎⁎^ denotes *p* < 0.01.

Compared to the CON group, dietary inclusion of CBS significantly increased serum levels of HDL cholesterol (*p* < 0.001), IgA, and IgG (*p* < 0.01), while significantly decreased serum concentrations of triglycerides and LDL cholesterol (*p* < 0.01) (Figure 2). No significant differences were observed in serum levels of total cholesterol or IgM among the three treatment groups.

### Antioxidant enzyme characteristics of serum and liver

**Fig. 3:**
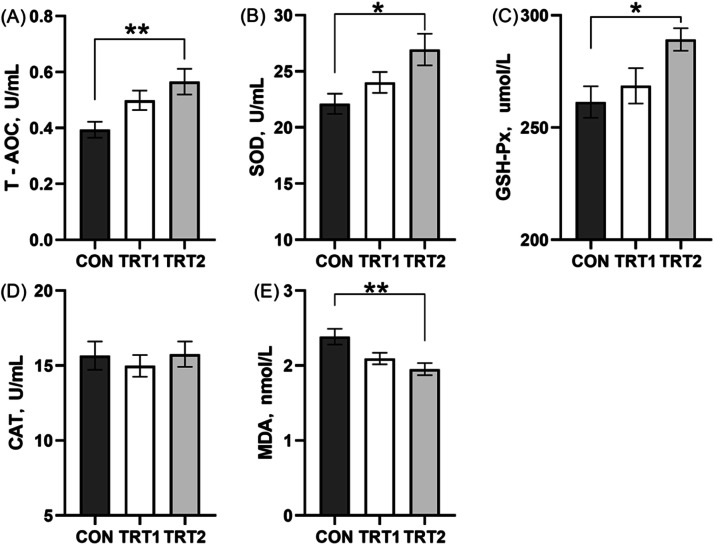
Serum antioxidant status on laying hens. (A) Total antioxidant. (B) Superoxide dismutase. (C) Glutathlone peroxidase. (D) Catalase. (E) Malondialdehyde. CON group, basal diet; TRT 1 group, basal diet+0.01 % combination of *B. subtilis* and *B. licheniformis*; TRT 2 group, basal diet+0.02 % combination of *B. subtilis* and *B. licheniformis*. Values are represented as mean ± SEM, *N* = 6. * denotes *p* < 0.05, ^⁎⁎^ denotes *p* < 0.01.

As shown in Figure 3, dietary inclusion of CBS significantly increased serum levels of T-AOC (*p* < 0.01), GSH-Px, and SOD (*p* < 0.05), while significantly decreased serum MDA (*p* < 0.01) levels compared to the CON group. No significant differences were observed in serum CAT levels among the treatment groups (*p* > 0.05).

**Fig. 4:**
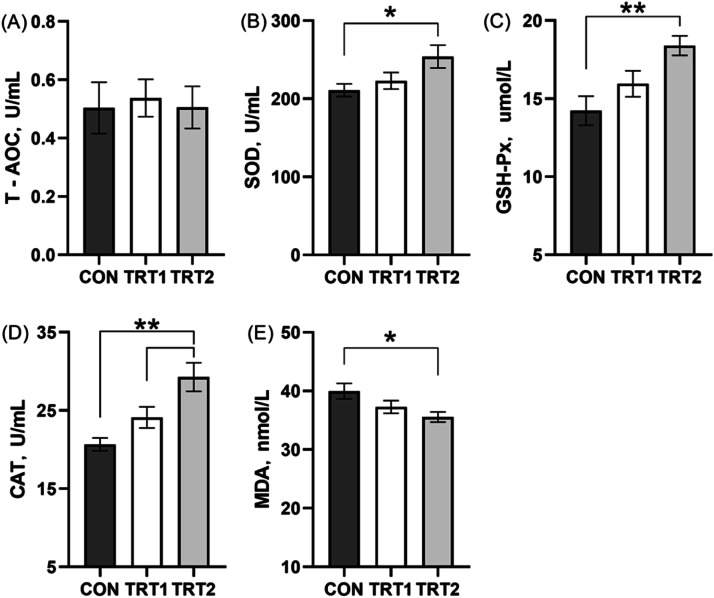
Liver antioxidant status on laying hens. (A) Total antioxidant. (B) Superoxide dismutase. (C) Glutathlone peroxidase. (D) Catalase. (E) Malondialdehyde. CON group, basal diet; TRT 1 group, basal diet+0.01 % combination of *B. subtilis* and *B. licheniformis*; TRT 2 group, basal diet+0.02 % combination of *B. subtilis* and *B. licheniformis*. Values are represented as mean ± SEM, *N* = 6. * denotes *p* < 0.05, ^⁎⁎^ denotes *p* < 0.01.

Furthermore, CBS supplementation significantly increased liver levels of SOD (*p* < 0.05), CAT, and GSH-Px (*p* < 0.01), while reducing liver MDA levels (*p* < 0.05) compared to the CON group. However, no significant difference was observed in liver T-AOC levels among the treatment groups (*p* > 0.05) (Figure 4).

### Intestinal morphology

**Fig. 5:**
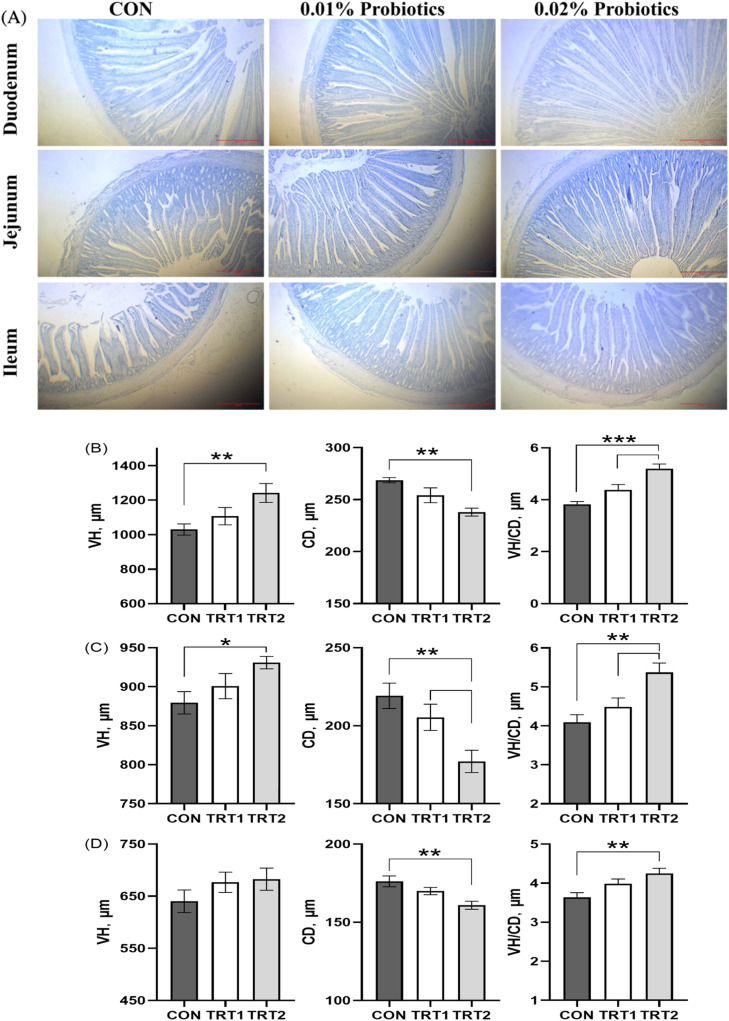
Effect of CBS supplementation on the intestinal morphology of laying hens. (A) Representative histological image of the duodenum, jejunum and ileum. (B) Duodenum. (C) Jejunum. (D) Ileum. CON group, basal diet; TRT 1 group, basal diet+0.01 % combination of *B. subtilis* and *B. licheniformis*; TRT 2 group, basal diet+0.02 % combination of *B. subtilis* and *B. licheniformis*. Values are represented as mean ± SEM, *N* = 6. * denotes *p* < 0.05, ^⁎⁎^ denotes *p* < 0.01.

As illustrated in Figure 5, dietary inclusion of CBS significantly increased VH in the jejunum (*p* < 0.01) and duodenum (*p* < 0.01), while significantly decreased CD in the duodenum, jejunum, and ileum (*p* < 0.01). The VH/CD ratios in the duodenum (*p* < 0.001), jejunum, and ileum (*p* < 0.01) were also significantly higher in the CBS-supplemented groups compared to the control.

### Caecal microbiota count

**Fig. 6:**
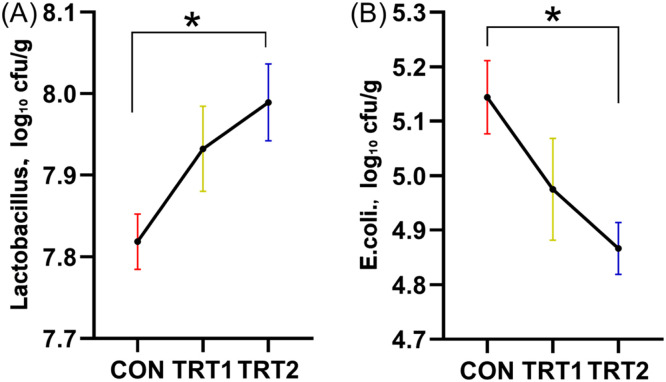
Excreta microbiota on laying hens. (A) *Lactobacillus*. (B) *E.coli*. CON group, basal diet; TRT 1 group, basal diet+0.01 % combination of *B. subtilis* and *B. licheniformis*; TRT 2 group, basal diet+0.02 % combination of *B. subtilis* and *B. licheniformis*. Values are represented as mean ± SEM, *N* = 6. * denotes *p* < 0.05, ^⁎⁎^ denotes *p* < 0.01.

As shown in Figure 6, hens fed diets supplemented with CBS exhibited a significantly higher (*p* < 0.05) *Lactobacillus* counts and significantly lower (*p* < 0.05) counts of *E. coli* compared to the CON group.

### Gas emission

**Fig. 7:**
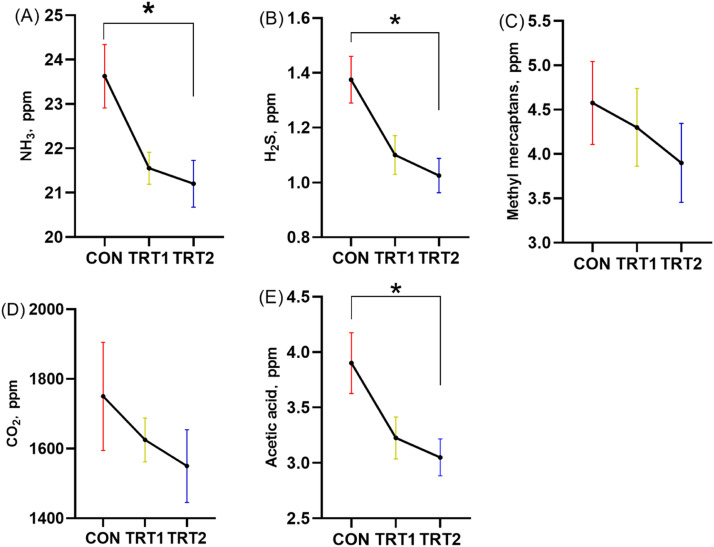
Gas emissions from laying hens. (A) Ammonia (NH_3_). (B) Hydrogen sulfide (H_2_S). (C) Total mercaptans. (D) Carbon dioxide (CO_2_). (E) Acetic acid. CON group, basal diet; CBS1 group, basal diet+0.01 % combination of *B. subtilis* and *B. licheniformis*; CBS2 group, basal diet+0.02 % combination of *B. subtilis* and *B. licheniformis*. Values are represented as mean ± SEM, *N* = 6. * denotes *p* < 0.05, ^⁎⁎^ denotes *p* < 0.01.

As shown in Figure. 7, diets containing CBS had lower (*p* < 0.05) gas emission of H_2_S, NH_3,_ and acetic acid concentrations compared to the CON group. However, the emission levels of methyl mercaptans and CO_2_ were not influenced among the treatment groups.

## Discussion

Enhancing productivity and egg quality in laying hens is critical for improving economic efficiency in the poultry industry (Arulnathan et al., 2024). Previous studies have demonstrated that CBS has been shown to have anti-inflammatory, antibacterial, and antioxidant properties (Romo-Barrera et al., 2021). In the present study, dietary supplementation with CBS increased egg production and reduced FCR in laying hens, while ADFI remained unchanged. Similarly, (Yang et al., 2020) reported a significant decrease in FCR in hens fed a diet supplemented with Bacillus licheniformis and Bacillus subtilis at 6.6 × 10⁵ : 3.3 × 10⁵ CFU/g. In contrast, (Forte et al., 2016b) found that supplementation with 0.05 % *B. subtilis* had no significant effect on the production performance of Hy-Line laying hens. Likewise, (Mahdavi et al., 2005) observed that adding fermented feed containing 4 % or 8 % probiotic formulations to Hy-Line Brown hens had no effect on growth performance. In addition, (Upadhaya et al., 2019) reported no significant effect of 500g/t of mixed probiotics on egg weight, Haugh unit and albumen quality. These discrepancies may be attributed to differences in the age of the birds, environmental conditions, and the hens’ adaptation to CBS supplementation across studies.

Egg production and egg quality were assessed during the laying phase (weeks 24 to 35). CBS supplementation improved egg production, consistent with findings by (Upadhaya et al., 2019), who reported increased egg production in Hy-Line laying hens fed diets containing 500g/t of mixed probiotics. Similarly, (Abdelqader et al., 2013) observed improved egg production in hens supplemented with 2.3 × 10⁸ CFU/g of *Bacillus* probiotics. (Lei et al., 2013) also reported enhanced egg production following dietary inclusion of *B. licheniformis.* However, in this study, the significant improvements were observed only in weeks 6 and 7, which may be related to a delayed effect as the birds gradually adapted to the probiotic supplementation. Probiotics have been shown to support digestive enzyme activity and nutrient utilization (Sen et al., 2012; Das et al., 2022). After peak production, egg quality typically declines, characterized by larger, broken eggs, poorer eggshell quality, and reduced albumen height (Saleh et al., 2019). Likewise, (Yang et al., 2020) found that dietary *Bacillus* species increased eggshell thickness, Haugh unit, and albumen quality. (Lei et al., 2013) reported that 0.01 % and 0.03 % 2 × 10^10^ CFU/g of *B. licheniformis* supplementation enhanced eggshell thickness and strength compared to controls. These improvements in eggshell properties have been attributed to probiotics’ ability to enhance metabolic processes and calcium digestibility (Abdelqader et al., 2013). Our study showed that CBS supplementation significantly improved Haugh unit, albumen height, eggshell thickness, and egg strength in laying hens, likely due to the antioxidant properties of probiotics, which may improve albumen quality by reducing lipid and protein oxidation. Laying hens exposed to oxidative stress from environmental and physiological factors often experience declines in production performance. The antioxidant enzymes GSH-Px and SOD reduce oxidative stress by neutralizing excess free radicals in the body (Zhang et al., 2011; Ighodaro et al., 2018). Together, SOD and GSH-Px catalyze the breakdown of intracellular superoxide anions and hydrogen peroxide. In vivo studies suggest that CAT functions similarly to GSH-Px. The T-AOC serves as a key indicator of the body’s overall non-enzymatic antioxidant defense system (Bartosz, 2010). MDA is a biomarker of oxidative stress, primarily resulting from lipid peroxidation (Schroers et al., 2024). Results from this study revealed increased serum and liver antioxidant markers in the probiotic-supplemented groups compared to controls, indicating that probiotic supplementation enhances antioxidant activity in laying hens.

*B. subtilis* can prevent the reabsorption of bile salts by converting them into secondary bile acids. Additionally, it synthesizes esterases that facilitate the conversion of free fatty acids into esterified forms, which differ from triglycerides within the gut. As a result, the absorption of cholesterol and triglycerides from the bloodstream into body cells is likely reduced. In our study, serum triglyceride and LDL cholesterol levels were significantly lower in the CBS group. Furthermore, studies have demonstrated that the addition of 150 mg/kg of probiotics plays a crucial role in intestinal lipid metabolism (Mohan et al., 1995). Serum immunoglobulins (IgM, IgA, and IgG), produced by B cells, serve as essential indicators of humoral immune function and reflect an animal’s ability to resist various diseases (Balan et al., 2019; Xu et al., 2021). Previous studies have shown that dietary supplementation with 4 × 10^10^ CFU/kg of *B. subtilis* (Bai et al., 2017) and *B. licheniformis* (Fazelnia et al., 2021) improves serum IgG and IgA levels in broilers. Our findings align with these results, supporting the conclusion that CBS supplementation enhances IgG and IgA concentrations in laying hens, thereby improving immune function.

Measures of intestinal morphology—such as increased VH, reduced CD, and higher VH/CD ratio—indicate improved nutrient absorption by increasing the intestinal surface area. Goblet cells in the intestinal villi and crypts secrete mucin that inhibits pathogen adhesion to the intestinal epithelium, serving as a markers of gut health (Jha et al., 2020). (Forte et al., 2016a) reported increased VH and VH/CD ratios in the jejunum, ileum, and duodenum of laying hens supplemented with 0.05 % *B. subtilis*. (Bromfield et al., 2024) found that dietary inclusion 500 g/t of *Bacillus* probiotics reduced CD in broilers. In this study, CBS supplementation significantly increased VH and VH/CD ratio while reducing CD in the small intestine, likely due to improved nutrient digestibility.

Living microorganisms utilize nutrients and energy for growth and reproduction. In our study, CBS inclusion significantly improved the digestibility of dry matter and nitrogen compared to the CON group. Earlier studies by (Priest, 1977) and (Carlisle et al., 1989) reported that complex probiotics improves nutrient digestibility by producing extracellular enzymes such as α-amylase and proteases. Complex probiotics supplementation has been shown to enhance nutrient digestibility in broilers (Zaghari et al., 2020; Bromfield et al., 2024) and pigs (Lan et al., 2019; Kim et al., 2022). (Guo et al., 2018) observed that *B. subtilis* improved dry matter and nitrogen digestibility in laying hens, consistent with findings from (Lei et al., 2013) who reported similar effects with *B. licheniformis*. (Oladokun et al., 2023) also found that CBS improved VH and VH/CD while reducing CD in chickens, further supporting its role in enhancing nutrient absorption.

The gastrointestinal tract of hens plays a vital role in digestion and nutrient absorption and hosts more than 1,000 bacterial species (Bernard et al., 2024). *Bacillus*-based probiotics enhance gut health by modulating immune responses (Scharek et al., 2007; Schierack et al., 2007), stimulating intestinal cell proliferation (Babinska et al., 2005), and improving nutrient transport across enterocytes (Walsh et al., 2012). In our study, CBS inclusion significantly increased *Lactobacillus* counts and reduced *E. coli* populations. Similarly, (Upadhaya et al., 2019) reported a significant increase in cecal *Lactobacillus* and a reduction in *E. coli* when CBS was included at 200–500 g/ton. (Lan et al., 2019) and (Kim et al., 2022) also observed that complex probiotics reduced excreta *E. coli* and improved *Lactobacillus* levels in pigs. However, (Hu et al., 2021) reported no significant effect of CBS on cecal bacterial counts, which may be attributed to differences in product composition, supplementation levels, and farm hygiene conditions.

Noxious gases such as NH₃, H₂S, methyl mercaptans, CO₂, and acetic acid released from poultry production pose environmental and health concerns (Muniyappan et al., 2022a). NH₃, in particular, contributes to soil and water acidification and nitrogen deposition in ecosystems. Previous studies suggest a strong relationship between gut microbiota, nutrient retention, and the release of harmful gases from excreta. (Ferket et al., 2002) reported that the abundance of pathogenic gut bacteria influences gas emissions in livestock. Therefore, reducing noxious gas emissions through proper management and nutritional strategies is essential for sustainable poultry production (Muniyappan et al., 2022b). In our study, CBS supplementation at 0.01 % and 0.02 % significantly reduced emissions of H₂S, NH₃, and acetic acid in laying hens. These findings are consistent with those of (Upadhaya et al., 2019), who observed reduced levels of NH₃ and H₂S following complex probiotics supplementation. Complex probiotics effectively reduced excreta emissions of NH₃ and H₂S through multiple mechanisms, including improving protein and sulfur utilization, modulating gut microbiota composition, suppressing the activity of gas-producing harmful bacteria, and enhancing intestinal health—such as increasing villus height and the VH/CD ratio. These effects collectively support the development of environmentally sustainable poultry production systems.

## Conclusion

In conclusion, supplementing laying hen diets with 0.02 % CBS significantly improved production performance and egg quality, enhanced immune function and antioxidant status, promoted improved intestinal morphology intestinal morphology, increased nutrient digestibility, modulated cecal microbiota, and reduced emissions of harmful excreta gases. These results underscore the potential of CBS as an effective probiotic feed additive to support the overall health and productivity of laying hens.

## CRediT authorship contribution statement

**Wei Han Zhao:** Writing – review & editing, Writing – original draft, Software, Formal analysis, Data curation, Conceptualization. **Madesh Muniyappan:** Writing – review & editing, Writing – original draft, Methodology, Investigation, Data curation, Conceptualization. **Shan Chuan Cao:** Writing – review & editing, Writing – original draft, Visualization, Validation, Supervision, Investigation, Conceptualization. **In Ho Kim:** Writing – review & editing, Writing – original draft, Visualization, Supervision, Software, Project administration, Methodology, Investigation, Funding acquisition, Conceptualization.

## Disclosures

The authors declare that they have no competing interests.
